# Optimal Brassinosteroid Levels Are Required for Soybean Growth and Mineral Nutrient Homeostasis

**DOI:** 10.3390/ijms22168400

**Published:** 2021-08-05

**Authors:** Ling Cheng, Man Li, Wanling Min, Mengke Wang, Rongqing Chen, Wenfei Wang

**Affiliations:** 1 College of Life Sciences, Fujian Agriculture and Forestry University, Fuzhou 350002, China; hbsdchengling@fafu.edu.cn (L.C.); NicoleStudy@fafu.edu.cn (M.W.); RongqingChen@fafu.edu.cn (R.C.); 2 College of Resources and Environment, Fujian Agriculture and Forestry University, Fuzhou 350002, China; LiMan@fafu.edu.cn (M.L.); 3200537063@fafu.edu.cn (W.M.)

**Keywords:** brassinosteroid, soybean, nutrient, transcriptome analysis, transporter

## Abstract

Brassinosteroids (BRs) are steroid phytohormones that are known to regulate plant growth and nutrient uptake and distribution. However, how BRs regulate nutrient uptake and balance in legume species is not fully understood. Here, we show that optimal BR levels are required for soybean (*Glycine max* L.) seedling growth, as treatments with both 24-epicastasterone (24-epiCS) and the BR biosynthesis inhibitor propiconazole (PPZ) inhibit root growth, including primary root elongation and lateral root formation and elongation. Specifically, 24-epiCS and PPZ reduced the total phosphorus and potassium levels in the shoot and affected several minor nutrients, such as magnesium, iron, manganese, and molybdenum. A genome-wide transcriptome analysis identified 3774 and 4273 differentially expressed genes in the root tip after brassinolide and PPZ treatments, respectively. The gene ontology (GO) analysis suggested that genes related to “DNA-replication”, “microtubule-based movement”, and “plant-type cell wall organization” were highly responsive to the brassinolide and PPZ treatments. Furthermore, consistent with the effects on the nutrient concentrations, corresponding mineral transporters were found to be regulated by BR levels, including the *GmPHT1*s, *GmKT*s, Gm*VIT2*, *GmZIP*s, and *GmMOT1* genes. Our study demonstrates that optimal BR levels are important for growth and mineral nutrient homeostasis in soybean seedlings.

## 1. Introduction

Brassinosteroids (BRs) are a group of steroid phytohormones, which have critical effects on cell elongation and diverse developmental and physiological processes [1,2]. BRs are recognized by their leucine-rich repeat (LRR) transmembrane receptor-like kinase BRI1 (BRASSINOSTEROID-INSENSITIVE 1) and co-receptor BAK1 (BRI1-ASSOCIATED RECEPTOR KINASE 1). Through a signaling cascade of phosphorylation and de-phosphorylation, the signal is transduced to the BZR (BRASSINAZOLE-RESISTANT) family of transcription factors and regulates the expression levels of thousands of downstream BR-responsive genes [2].

Recently, studies in legume species revealed that BRs are involved in photomorphogenesis and skotomorphogenesis, root development, flowering, pod development, nodulation, gravitropism, senescence, and response to drought stresses [3,4,5,6]. Soybean fresh weight and growth were decreased after a root-applied epibrassinolide treatment [3]. Brassinolide (BL) was found to promote hypocotyl and epicotyl elongation under light, but inhibit epicotyl development in the dark [3]. Low levels of BRs caused by treatment with the biosynthesis inhibitor, brassinazole, resulted in an increase of the nodule number and suppressed the pod growth in soybean [7,8]. However, the BR-deficient mutants, *lk* and *lkb*, and the receptor mutant, *lka*, displayed a reduced nodulation in pea (*Pisum sativum*), and the *mtbri1* mutant displayed a decreased nodulation and symbiotic nitrogen fixation in *M. truncatula* [9], which indicates the complex regulation on nodulation by BRs [10]. In addition, BL treatment repressed the negative gravitropism of shoot and delayed the leaf senescence in soybean [3]. BRs improved the photosynthesis and increased the chlorophyll content in soybean when the foliar was applied before drought stress [6]. When exogenous BRs and the specific BR biosynthesis inhibitor, propiconazole (PPZ), were applied to soybean and other plants, transcriptional analyses revealed a wide range of cellular activities and biological processes, such as cell wall organization, carbohydrate metabolism, and cellular response to oxidative stress [11,12]. The BR biosynthesis genes, *GmCPDs* (*CONSTITUTIVE PHOTOMORPHOGENESIS AND DWARFISM*), are shown to be involved in flowering control and floral development [13]. From soybean and medicago, the BR receptors, *GmBRI1* and *MtbBRI1*, were cloned and functionally analyzed in Arabidopsis [9,14] and were able to restore the dwarf phenotype of a weak allele of the *AtBRI1* mutant, *bri1-5*. Moreover, several soybean *BZR* family genes were analyzed, and *GmBZL2* and *GmBZL3* were reported to play conservative roles in the BR signaling pathway [15,16]. GmBEHL1, a close ortholog of AtBEH1 (BES1/BZR1 HOMOLOG 1), was reported to interact with the NNC1 (NODULE NUMBER CONTROL 1) protein to negatively regulate soybean nodulation [5]. 

BRs are reported to play important roles in nutrient uptake and distribution. Phosphorus (P) deficiency reduces the endogenous BR amount and the nuclear localization of BZR1 and BES1 (BRI1-EMS-SUPPRESSOR 1) proteins in the elongation zone of the Arabidopsis root [17]. In addition, owing to the tight interplay between BR and iron signaling pathways, it was reported that BR activity controls the iron (Fe) accumulation in expanding cells, while low iron promotes root growth by activating brassinosteroid signaling. Interestingly, BR regulates iron homeostasis in a complex way, and the exogenous application of 24-epibrassinolide increased and decreased the Fe contents in rice roots and shoots, respectively, under both Fe-deficient and Fe-sufficient conditions [4]. Recently, BR treatment was able to alleviate the growth inhibition caused by Ca(NO_3_)_2_, manganese (Mn), salt, and other ion stresses, and it regulated the homeostasis of several mineral nutrients [18,19,20,21]. In 2015, Yuan et al. reported that the foliar spray of 24-epibrassinolide significantly alleviated the inhibition of several nutrients, such as P, potassium (K), nitrogen (N), sodium (Na), magnesium (Mg), Fe, and Mn uptake in Ca(NO_3_)_2_-stressed cucumber plants [21]. BR improved the Ca^2+^/Na^+^ and K^+^/Na^+^ ratio of wheat cultivars under salt stress by enhancing the uptake of Ca^2+^ and K^+^ and reducing that of Na^+^ [18]. 24-epibrassinolide treatment promoted the uptake of K^+^, Ca^2+^, Mg^2+^, and NO_3_^-^ in salt-stressed canola (*Brassica napus* L.) [20].

In the present study, we investigated the function of BR on the growth and nutrient homeostasis in soybean and further performed a RNA-sequencing analysis of the BR responsive transcriptomes. Our results demonstrate an essential role of BR in the regulation of nutrient homeostasis and seedling growth in soybean through regulating hundreds of downstream genes, including mineral transporters.

## 2. Results

### 2.1. Optimal BR Levels Are Required for Soybean Growth

**Figure 1:**
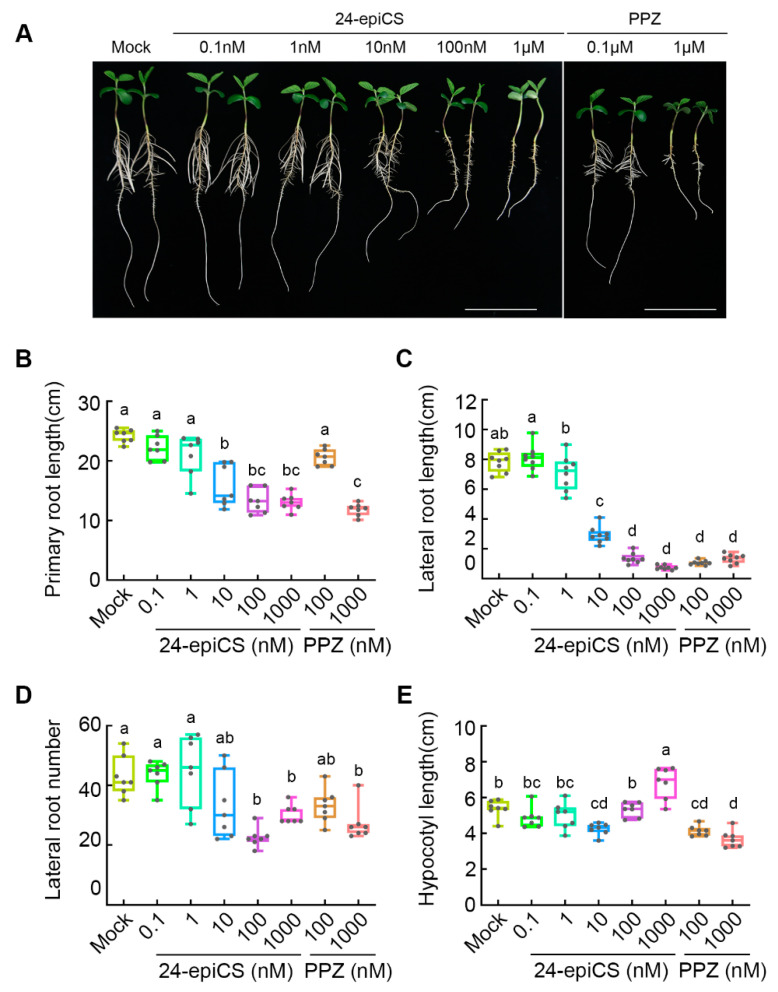
Soybean seedling under 24-epicastasterone (24-epiCS) and propiconazole (PPZ) treatments. (**A**) Morphology of whole seedlings grown in different concentrations of 24-epiCS and PPZ for 4 days (scale bar = 10 cm). (**B**–**E**) Quantitation of the primary root length (**B**), lateral root length (**C**), lateral root number (**D**), and hypocotyl length (**E**) after different 24-epiCS and PPZ treatments. Different letters indicate significant differences between the different treatments (*n* = 7, *p* < 0.05, one-way analysis of variance (ANOVA)).

To investigate the function of brassinosteroid in soybean growth, we applied a native BR precursor with high bioactivity, 24-epicastasterone (24-epiCS), and PPZ [22] to the hydroponically grown soybean seedlings for 4 days (Figure 1A). The application of 24-epiCS (0.1 nM, 1 nM, 10 nM, 100 nM, and 1000 nM) reduced the primary root length (Figure 1B), lateral root length (Figure 1C), and numbers (Figure 1D) in a concentration-dependent manner. The application of 1 μM of 24-epiCS decreased the primary root length from 24.18 cm to 13.07 cm (Figure 1B) and dramatically decreased the lateral root length from 7.85 cm to 0.75 cm (Figure 1C). Contrary to what was observed in the root, the hypocotyl length was increased by the high concentration of 24-epiCS (1 μM) from 5.38 cm to 6.84 cm (Figure 1A,E), which is consistent with a previous report [3]. With the PPZ treatment inducing an endogenous BR reduction, both the shoot and root growth of soybean were inhibited (Figure 1A). High-concentration PPZ significantly reduced the primary root length, lateral root length, and hypocotyl length and slightly reduced the lateral root number (Figure 1B–D). Collectively, these data demonstrate that BR is essential for soybean growth, and optimal endogenous BR levels are required for the root growth.

### 2.2. BR Regulates the Homeostasis of Nutrient Elements

**Figure 2:**
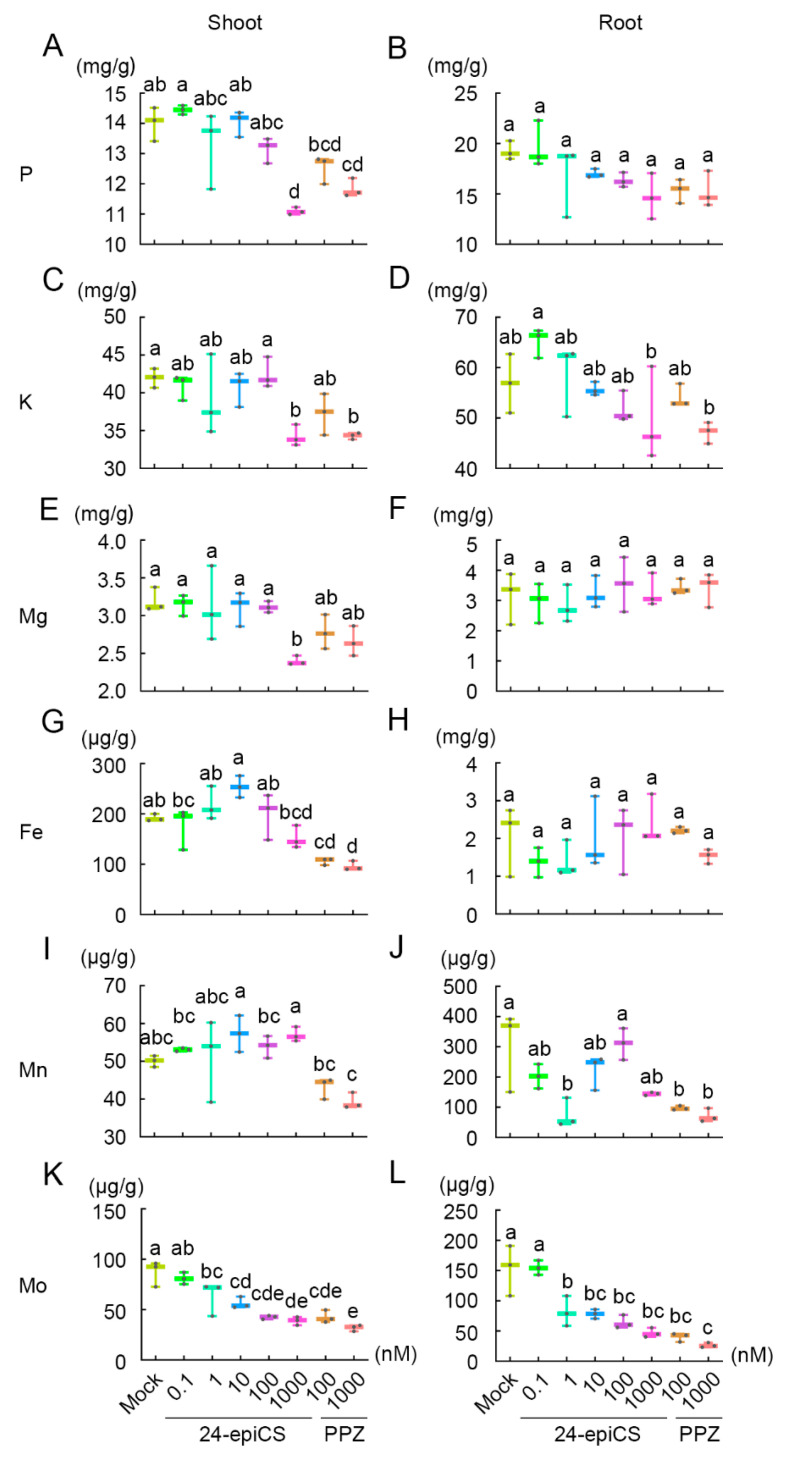
Nutrient element concentration after 24-epiCS and PPZ treatments. Soybean seedlings were treated with different concentrations of 24-epiCS and PPZ, and the shoot and root parts were harvested for inductively coupled plasma mass spectrometry (ICP-MS), P (**A**,**B**), K (**C**,**D**), Mg (**E**,**F**), Fe (**G**,**H**), Mn (**I**,**J**), Mo (**K**,**L**). Three biological replicates were used for each sample. Different letters indicate significant differences between the different treatments (*n* = 3, *p* < 0.05, one-way ANOVA).

Previous studies showed that BR modulates the uptake of several mineral nutrients and metabolism [18,19,20,21]. To determine whether BR affects the nutrient uptake in soybean, we measured the element concentration of the abovementioned treatments with 24-epiCS and PPZ applied to soybean seedlings by inductively coupled plasma mass spectrometry (ICP-MS), with shoot and root tested separately (Table S1). Compared with mock plants, plants treated with 24-epiCS and PPZ showed reduced total phosphorus and potassium levels in the shoot, but not the root (Figure 2A–D). In the shoot, high-concentration 24-epiCS (1.0 μM) and PPZ (1.0 μM) treatments reduced the phosphorus concentrations from 19.253 mg/g (P/fresh weight) to 14.723 mg/g and 15.275 mg/g, respectively (Figure 2A). Similarly, treatments with 1.0 μM of 24-epiCS and 1.0 μM of PPZ decreased the total K levels from 41.986 mg/g to 34.235 mg/g and 34.298 mg/g, respectively (Figure 2C).

In addition to the macronutrients, several minor nutrients were affected by the BR levels (Figure 2E–L). When 1 μM of exogenous 24-epiCS was applied, magnesium (Mg) was decreased in the shoot parts (Figure 2E). By contrast, the PPZ treatment (0.1 μM and 1.0 μM) led to a substantial decrease in the iron (Fe) level in the shoot specifically, while the increased 24-epiCS level had no significant effects (Figure 2G). On the other hand, the manganese (Mn) level in the root was found to be reduced after the PPZ treatment (0.1 μM and 1.0 μM) (Figure 2J). Intriguingly, the concentration of the molybdenum (Mo) element was sensitive to the increased 24-epiCS and PPZ levels in both the shoot and root (Figure 2K,L). The Mo concentrations were decreased by both the 24-epiCS and PPZ treatments in dose-dependent manners (Figure 2K,L). Taken together, these results suggest that BR levels are crucial for the homeostasis of nutrient elements.

### 2.3. Transcriptomic Changes in the Soybean Root Tip by BR

**Figure 3:**
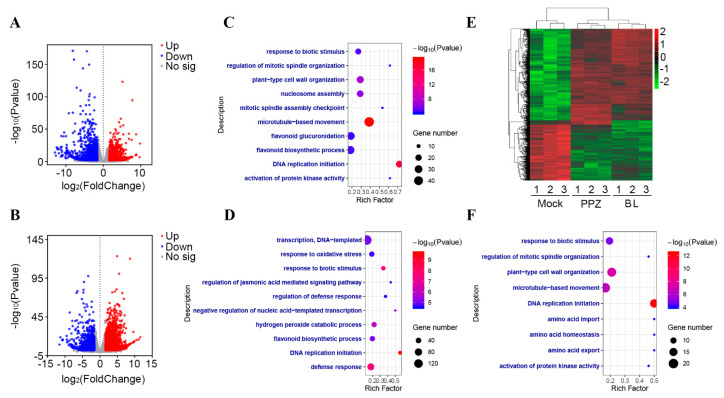
Transcriptome-wide analysis of BR- and PPZ-responsive genes. (**A**,**B**) Volcano plot of differentially expressed genes after the BL (**A**) and PPZ (**B**) treatments. Red dots indicate upregulated genes, and blue dots indicate downregulated genes, with a fold change ≥ 3 or ≤ -3 as the significance threshold and adjusted *p*-value ≤ 0.01. (**C**,**D**) Gene ontology analysis of BL- (**C**) and PPZ-regulated (**D**) genes. (**E**) Heat map of the expression profiles of the genes regulated by BL, PPZ, and a mock solution. The color scale at the top represents the average log signal intensity values. (**F**) Gene ontology analysis of BL and PPZ co-regulated genes.

To further investigate BRs’ function in root growth and nutrient balance, we carried out RNA-seq analyses of the root tip (1 cm) of soybean seedlings grown in a hydroponic medium. Treatments with 0.01 nM of BL and 0.1 μM of PPZ applied to the root tips were compared with mock-treated samples. Differentially expressed genes, after the BL and PPZ treatments (fold change ≥3 or ≤−3, adjusted *p* value ≤ 0.01), were identified by statistical analysis and are displayed using volcano plots (Figure 3A,B). Compared with the mock treatment, BL increased the expression levels of 2257 genes and decreased the expression of 1517 genes in the soybean root tip (Figure 3A) (Table S2). Gene ontology (GO) analysis showed that the most enriched four categories were “microtubule-based movement”, “DNA replication initiation”, “plant-type cell wall organization”, and “nucleosome assembly” (Figure 3C). PPZ treatment increased 2882 genes and decreased 1391 genes (Figure 3B). Gene ontology (GO) analysis showed that the most enriched four categories were “response to biotic stimulus”, “DNA-replication”, “defense response”, and “hydrogen peroxide catabolic process” (Figure 3D). To better understand the BR-responsive expression profiles, a heat map of the differentially expressed genes was built with BL, PPZ, and mock-treated samples. Overall, about 97.08% (1797) of the 1851 co-regulated genes were affected in the same way by the BL and PPZ treatments (Figure 3E), with a pairwise comparison Pearson correlation coefficient of 0.8987. Such similar genomic effects were consistent with the altered root growth phenotype and nutrient concentrations (Figure 1 and Figure 2). Furthermore, GO analysis showed that these co-regulated genes were closely related to biological processes related to “DNA-replication”, “microtubule-based movement”, and “plant-type cell wall organization” (Figure 3F).

### 2.4. Quantitative RT-PCR Analysis of Mineral Transporter Genes

**Figure 4:**
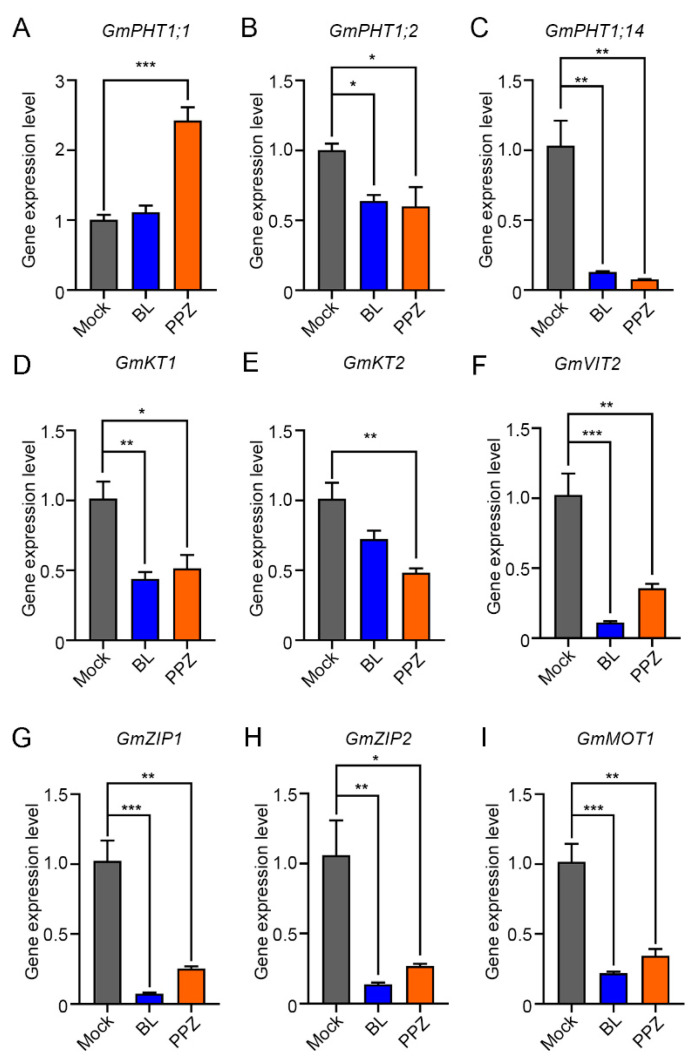
qRT-PCR analysis of BR-responsive mineral transporter genes. Expression levels of *GmPHTs* (**A**–**C**), *GmKTs* (**D**,**E**), *GmVIT2* (**F**), *GmZIPs* (**G**,**H**), and *GmMOT1* (**I**) genes after the BL and PPZ treatments. This experiment was biologically repeated three times. The error bars indicate the standard deviation and the significant differences from the mock samples are marked (*n* = 3, * *p* < 0.05, ** *p* < 0.01, *** *p* < 0.001, Student’s *t*-test).

Mineral transporters were reported to play important roles in the nutrient acquisition and redistribution [23]. Several transporter genes were found to be differentially expressed after BL or PPZ treatments in the RNA-seq analysis (Table S2), and quantitative RT-PCR assays were performed to validate the expression levels (Figure 4). Three *PHT1* (*PHOSPHATE TRANSPORTER 1*) family genes were found to be responsive to the BR levels. The expression level of *GmPHT1;1* was reduced by the BL treatment only, while the *GmPHT1;2* and *GmPHT1;14* genes were repressed by both the BL and PPZ treatments (Figure 4A–C). In addition, two potassium (K) transporter genes, named *GmKT1* (*Glyma.08G086300*) and *GmKT2* (*Glyma.13G170700*), were downregulated by both the BL and PPZ treatments (Figure 4D,E), which have been functionally characterized as potassium transporters for Na^+^/K^+^ ratio regulation [24]. Similarly, *Glyma.08G181900* (named *GmVIT2*, *VACUOLAR IRON TRANSPORTER 2*) and two *GmZIP* (*ZINC REGULATED TRANSPORTER/IRON-REGULATED TRANSPORTER-RELATED PROTEIN*) genes, *Glyma.13G338300* (named *GmZIP1*) and *Glyma.08G328000* (named *GmZIP2*), were found to be downregulated by BL and PPZ (Figure 4D). *Glyma.17G203200* (named *GmMOT1*), encoding a high-affinity molybdate transporter in soybean, was downregulated by the BL and PPZ treatments, which is consistent with the decreased Mo concentration in both the shoot and root. These results indicate that BR may regulate nutrient concentration by controlling the downstream responsive genes, including mineral transporter genes.

## 3. Discussion

### 3.1. BR May Regulate Nutrient Concentration through the Transcriptional Regulation of Mineral Transporter Genes

Transcriptome analyses revealed that BRs regulate thousands of downstream genes via BZR transcription factors, including genes related to cell wall organization, cytoskeleton organization, carbohydrate metabolism and cellular responses to oxidative stress [2,11,12]. Our RNA-seq analysis revealed that BRs regulate similar downstream genes in soybean root tips. Previous studies reported that BRs regulate the nutrient concentration in plants, especially under stress conditions [4,18,20,21]. However, we did not find clear gene group-related nutrients from the GO analysis, probably because only a small number of genes contribute to the nutrient balance. To investigate the possible regulation details, we focus on the differentially expressed transporter genes from our RNA-seq data. In Arabidopsis, the expression levels of *AtPHT1;4* and *AtPHT1;9* were not affected by the core transcription factor BZR1 activity [17]. Consistent with the decreased P concentration, the *GmPHT1;2* and *GmPHT1;14* genes were downregulated by both the BL and PPZ treatments in the soybean root tip (Figure 4) under our treatments, which may have contributed to the observed change in the P concentration. *GmPHT1;14* was found to be expressed in the root specifically and dramatically induced by the low phosphate condition [25]. *GmPHT1;1,* also called *GmPT7*, was reported to be highly expressed in the root and nodule, thus enhancing the symbiotic N_2_ fixation and yield in soybean [26]. The expression level of *GmPHT1;1* was increased when reducing the BR by PPZ, indicating that the regulation of *GmPHT1;1* does not simply match the BR levels. One possible explanation for this is that the main responsive transporter is *GmPHT1;14*, and there might be a compensatory increase of *GmPHT1;1*, as the *GmPHT1;14* expression level is much higher in the root [25]. Another possible reason is that the regulation of *GmPHT1;1* from PPZ may take place on the post-translational level, and the reduction in the mRNA level was a feedback regulation. Further analysis is required to uncover the mechanism in detail.

Exogenous 24-epibrassinolide was found to reduce the K^+^ efflux via depolarization-activated K^+^ channels under salt stress [27], while our data show that both the BR and PPZ treatments reduced the K amount and expression levels of two *KT* genes. The soybean yield and quality were reported to be seriously decreased under Mo-deficient conditions, while the application of Mo promotes shoot and root growth and the photosynthesis rate [28]. In our experiment, the Mo concentration was reduced by increasing and decreasing the BR levels, which is consistent with the inhibition of the root growth (Figure 2). As *GmMOT1* was found to be a BR-responsive gene, BR may regulate the Mo uptake by regulating the expression of Mo transporters at the transcriptional level. Further research on the mechanism of the interaction between Mo and BR needs to be carried out.

### 3.2. BR Regulates the Nutrient Distribution between the Shoot and Root

Mineral nutrients are taken up by the roots and are transported to the shoot by the xylem. The distribution of the mineral nutrients between the shoot and root is believed to be determined by the developmental status and environmental conditions to optimize growth and survival. It is worth noting that, when the BR or PPZ treatments were applied to the root part, the concentrations of P, K, Fe, and Mg were affected by the BR level in the shoot only. On the other hand, BR is required for the normal Mn concentration in the root, while its level did not trigger dramatic differences in the shoot (Figure 2). Previously, BR was reported to regulate Fe contents, with different trends for the shoot and root of rice, respectively [4], while BR promotes the Fe concentration in the shoot of Ca(NO_3_)_2_-stressed cucumber plants [21], which suggests that the BR regulation of the Fe concentration may be affected by different environmental conditions in different species. In 2020, Trevisan et al. reported that eBL regulated the expression of several mineral transporter genes in opposite transcriptional profiles in the root and aerial parts of maize plants [29]. Interestingly, BRs are not believed to be transported over long distances [30,31], suggesting that other long-distance communication signals are required for nutrient coordination between the shoot and root.

## 4. Conclusions

Our data show that optimal BR levels are required for soybean seedling growth, especially the root growth. Both a lack and excess of BRs would disturb the primary root elongation, lateral root formation, and elongation. Exogenous treatments with 24-epiCS and PPZ reduce the concentration of P and K in the shoot part specifically. 24-epiCS decreased the Mg concentration in the shoot part, while the PPZ treatment decreased the Fe and Mn concentrations in the shoot and root, respectively. Moreover, both 24-epiCS and PPZ reduced the Mo concentration in a dose-dependent manner. The current results indicate a key role of the steroid phytohormone, brassinosteroid, in the balance of nutrient homeostasis and growth. Our findings will provide the basis for further studies on BR-responsive genes and the molecular mechanism of nutrient uptake and distribution in soybean.

## 5. Materials and Methods

### 5.1. Soybean Growth and Phenotype Analysis

The soybean (*Glycine max*) genotype, YC03-3, was used in this study. For the phenotype analysis, YC03-3 was separately treated with epicastasterone (24-epiCS) and PPZ. After 3 days of germination, the seedlings were grown in a full-strength nutrient solution, supplemented with 0.1 nM, 1 nM, 10 nM, 100 nM, or 1000 nM of 24-epiCS and 100 nM or 1000 nM of a PPZ solution for 4 days. The primary root length, hypocotyl length, lateral root length, and lateral root number of seven seedlings were measured by ImageJ (bundled with Java 1.8.0_172) (https://imagej.nih.gov/ij/). All the collected data were analyzed by GraphPad Prism (v8.0.2.263) (https://www.graphpad.com/scientific-software/prism/) using one-way analysis of variance (ANOVA).

### 5.2. Element Concentration Measurement

The leaf and root tissues after the 24-epiCS and PPZ treatments were used for the total element quantitative measurements. After drying at 65 °C for 2 days, the samples were weighed and digested in concentrated H_2_SO_4_. The concentrations of the nutrient elements (K, Mg, Fe, Zn, B, Ca, Mn, Mo, and Al) were determined by ICP-MS (7900 Mass Spectrometer, Agilent, Santa Clara, CA, USA). The concentrations of P and N were determined by a Continuous Flow Analyzer (Skalar San^++^ system, The Netherlands).

### 5.3. RNA Isolation, Library Construction, and RNA Sequencing

Hydroponically grown root tips (1 cm from the root tip-end) were collected after the treatments with the mock solution, 0.01 nM of brassinolide, and 0.1 μM of PPZ. Nine samples (three biological replicates of the mock-, BL-, and PPZ-treated root tips) were used for the construction and sequencing of the mRNA library. The total RNA was extracted from each sample. A total amount of 1 µg RNA per sample was used for the sample preparations. Sequencing libraries were generated using the NEBNext UltraTM RNA Library Prep Kit for Illumina (NEB, Ipswich, MA, USA). Using poly-T oligo-attached magnetic beads, the mRNA was purified and fragmented into short pieces, which were thus used as templates for cDNA synthesis [32]. Finally, each library was sequenced by the Illumina Novaseq platform at the Novogene corporation (Tianjin, China). The RNA-seq raw data were deposited in the Gene Expression Omnibus (GEO) database (https://www.ncbi.nlm.nih.gov/geo/) as GSE175586.

### 5.4. RNA-Seq Reads’ Mapping and Differential Counting 

The reference genome and gene model annotation files were directly downloaded from Phytozome (https://phytozome.jgi.doe.gov/pz/portal.html). The index of the reference genome was built, and paired-end clean reads were aligned to the reference genome using Hisat2 v2.0.5 [33]. FeatureCounts v1.5.0-p3 was used to count the read numbers mapped to each gene [34]. Differentially expressed genes (DEGs) between the two sets of samples were identified using the DESeq2 R package [35,36]. The genes with a fold change ≥3 or ≤−3 and an adjusted *p*-value (padj) ≤ 0.01 were considered as significant DEGs.

### 5.5. Functional Annotation and Gene Ontology (GO) Enrichment

The DEGs were annotated for GO terms and categorized into biological process categories. The GO terms were obtained from the DAVID (version 6.8) platform (https://david.ncifcrf.gov/) [37]. The GO enrichment analyses were conducted using the R Programming Language, with a *p*-value ≤ 0.05 regarded as significantly enriched [35]. The Novogene tool (https://magic.novogene.com/) was used to perform bidirectional clustering analysis of all the different genes in the samples.

### 5.6. Primer Design and Validation of the RNA-Seq Data

The primer pairs for flanking sequences of each unique gene were designed automatically using DNAMAN 8 and are listed in Table S3. The qRT-PCR reactions were performed using the QuantStudio 6 Flex Real-Time PCR System, with the TaKaRa Real-time qPCR Master Mix Kit. The qRT-PCR experiments were performed with biological triplicates, and the relative gene expression level was calculated using the 2^−^^△△Ct^ method, with *GmUBI3* as the reference gene.

## Data Availability

The RNA-seq raw data were deposited in the Gene Expression Omnibus (GEO) database as GSE175586.

## Supplementary Materials

The following are available online at https://www.mdpi.com/article/10.3390/ijms22168400/s1.

## Abbreviations

24-epiCS	24-epicastasterone
BL	Brassinolide
BR	Brassinosteroid
BRs	Brassinosteroids
DEGs	Differentially expressed genes
GEO	Gene Expression Omnibus
GO	Gene ontology
ICP-MS	Inductively coupled plasma mass spectrometry
*KT*	*POTASSIUM (K) TRANSPORTER*
*MOT1*	*MOLYBDATE TRANSPORTER 1*
*PHT1*	*PHOSPHATE TRANSPORTER 1*
PPZ	Propiconazole
*VIT2*	*VACUOLAR IRON TRANSPORTER 2*
*ZIP*	*ZINC REGULATED TRANSPORTER/IRON-REGULATED TRANSPORTER-RELATED PROTEIN*
