# Supplementary material for: Optimal Brassinosteroid Levels Are Required for Soybean Growth and Mineral Nutrient Homeostasis
Source: Int J Mol Sci. 2021 Aug 5;22(16):8400. doi: 10.3390/ijms22168400 (PMC8395106; doi:10.3390/ijms22168400)
Supplement: Supplementary file 1 [file ijms-22-08400-s001.zip › Table S1 Concentrations of nutrient elements after the 24-epiCS and PPZ treatments.pdf]

Table S1. Nutrient elements concentration upon BR and PPZ treatment

		Mock		24-epiCS										PPZ			
				0.1 nM		1 nM		10 nM		100 nM		1000 nM		100 nM		1000 nM	
		Average (mg/g)	STDEV	Average (mg/g)	STDEV	Average (mg/g)	STDEV	Average (mg/g)	STDEV	Average (mg/g)	STDEV	Average (mg/g)	STDEV	Average (mg/g)	STDEV	Average (mg/g)	STDEV
**N**	shoot	61.248 ab	0.892	63.381 ab	1.678	63.556 a	3.396	62.708 ab	1.875	62.029 ab	0.835	57.814 b	1.421	63.835 a	1.724	61.762 ab	2.801
	root	40.695 a	2.220	42.928 a	1.168	40.939 a	6.313	40.352 a	2.114	39.394 a	2.744	43.842 a	9.850	45.711 a	3.207	50.213 a	2.208
**P**	shoot	14.013 ab	0.560	14.447 a	0.147	13.275 abc	1.275	14.033 ab	0.428	13.146 abc	0.420	11.094 d	0.124	12.520 bcd	0.457	11.840 cd	0.305
	root	19.253 a	0.928	19.659 a	2.313	16.760 a	3.528	17.015 a	0.418	16.354 a	0.720	14.723 a	2.260	15.344 a	1.191	15.275 a	1.782
**K**	shoot	41.986 a	1.270	40.900 ab	1.652	39.135 ab	5.349	40.728 ab	2.298	42.457 a	2.044	34.235 b	1.413	37.258 ab	2.732	34.298 b	0.426
	root	56.881 ab	5.849	65.220 a	2.909	58.477 ab	7.124	55.712 ab	1.323	51.878 ab	3.127	49.693 b	9.346	54.177 ab	2.307	47.183 b	2.132
**Mg**	shoot	3.195 a	0.159	3.147 a	0.138	3.122 a	0.494	3.109 a	0.226	3.114 a	0.075	2.401 b	0.062	2.779 ab	0.226	2.655 ab	0.198
	root	3.149 a	0.860	2.958 a	0.653	2.842 a	0.620	3.237 a	0.530	3.544 a	0.904	3.286 a	0.551	3.435 a	0.254	3.407 a	0.564
**Fe**	shoot	0.192 ab	0.007	0.176 bc	0.041	0.218 ab	0.033	0.254 a	0.021	0.199 ab	0.046	0.152 bcd	0.022	0.106 cd	0.007	0.096 d	0.009
	root	2.047 a	0.934	1.376 a	0.392	1.408 a	0.484	2.014 a	0.963	2.050 a	0.892	2.435 a	0.645	2.217 a	0.085	1.535 a	0.188

**Zn**	shoot	0.126	0.064	0.162	0.022	0.094	0.009	0.146	0.025	0.136	0.028	0.168	0.056	0.169	0.081	0.177	0.044
		a		a		a		a		a		a		a		a	
	root	0.051	0.015	0.048	0.007	0.154	0.129	0.089	0.047	0.062	0.000	0.075	0.006	0.101	0.038	0.096	0.031
		a		a		a		a		a		a		a		a	
**B**	shoot	0.062	0.010	0.058	0.011	0.036	0.009	0.034	0.017	0.048	0.019	0.055	0.039	0.058	0.018	0.063	0.028
		a		a		a		a		a		a		a		a	
	root	0.065	0.025	0.033	0.003	0.049	0.017	0.032	0.017	0.041	0.010	0.039	0.036	0.043	0.016	0.039	0.022
		a		a		a		a		a		a		a		a	
**Ca**	shoot	0.910	0.238	0.870	0.125	0.558	0.230	0.664	0.059	0.695	0.130	0.710	0.152	0.686	0.065	0.677	0.167
		a		a		a		a		a		a		a		a	
	root	0.435	0.062	0.407	0.057	0.501	0.045	0.490	0.022	0.336	0.072	0.356	0.025	0.530	0.108	0.500	0.088
		ab		ab		ab		ab		b		ab		a		ab	
**Mn**	shoot	0.050	0.001	0.053	0.000	0.051	0.011	0.057	0.005	0.054	0.003	0.057	0.002	0.043	0.003	0.039	0.002
		abc		ab		abc		a		ab		a		bc		c	
	root	0.304	0.133	0.202	0.040	0.076	0.048	0.221	0.056	0.310	0.052	0.144	0.005	0.097	0.007	0.071	0.023
		a		ab		b		ab		a		ab		b		b	
**Mo**	shoot	0.087	0.013	0.081	0.006	0.063	0.017	0.056	0.006	0.042	0.002	0.039	0.004	0.043	0.006	0.032	0.003
		a		ab		bc		cd		cde		de		cde		e	
	root	0.153	0.042	0.155	0.012	0.082	0.025	0.078	0.008	0.064	0.011	0.047	0.008	0.040	0.007	0.026	0.004
		a		a		b		bc		bc		bc		bc		c	
**Al**	root	1.099	0.618	0.650	0.190	0.678	0.218	1.092	0.607	1.095	0.640	1.301	0.507	1.306	0.019	0.732	0.250
		a		a		a		a		a		a		a		a	

3d after seed germination, seedlings were grown in a full-strength nutrient solution supplemented with 0.1 nM, 1 nM, 10 nM, 100 nM, or 1000nM 2,4-epicastasterone (24-epiCS) and 100 nM or 1000 nM propiconazole (PPZ) solution for 4 days. Leaf and root tissue of soybean seedlings after 24-epiCS and PPZ treatment were used for the total element quantitative measurements.
