# Supplementary material for: Optimal Brassinosteroid Levels Are Required for Soybean Growth and Mineral Nutrient Homeostasis
Source: Int J Mol Sci. 2021 Aug 5;22(16):8400. doi: 10.3390/ijms22168400 (PMC8395106; doi:10.3390/ijms22168400)
Supplement: Supplementary file 1 [file ijms-22-08400-s001.zip › Table S3 List of primers used for the qPCR analysis.pdf]

Table S3: List of primers used in this paper.

Gene locus	Primer name	Sequence (5’ to 3’)
Glyma.10G186500	GmPHT1;1 qF1	GTGAGTGATGGCGGGAGGAC
	GmPHT1;1 qR1	GATCATAGGCATCGGTGAAGAAT
Glyma.03G162800	GmPHT1;2 qF1	ACGCATATTTGTTGATTTCACTTG
	GmPHT1;2 qR1	CTCTGCACAAGTGGAAACAAGAGTC
Glyma.02G005800	GmPHT1;14 qF1	TTGGTTGTGACGTGTTTACACC
	GmPHT1;14 qR1	ACAAAAGTAAGAACCAAAAGCAAAC
Glyma.08G181900	GmVIT2 qF1	GGTTAAGTCCACTCTCAG
	GmVIT2 qR1	CTCCAAGTCCAAGATGAT
Glyma.17G203200	GmMOT1 qF1	TCCTGTGGGGATCTTAGGGG
	GmMOT1 qR1	GCTCAGGACTTTTCTGCATCC
Glyma.13G338300	GmZIP1 qF1	GCTTATATGCTGGCTTCA
	GmZIP1 qR1	CTGTTGCTATTCCTGGTAA
Glyma.08G328000	GmZIP2 qF1	TACTTCTCAATCACAACA
	GmZIP2 qR1	CAACTGAATGGACATATC
Glyma.08G086300	GmKT1 qF1	GGCACAGATAGAGTTAGTT
	GmKT1 qR1	GCTTCCATACTTCCAGAT
Glyma.13G170700	GmKT2 qF1	AGGATATTCAGCATTCAGA
	GmKT2 qR1	CTTCACCATTGTCATCAG
Glyma.20G141600	GmUBI3-qF	GTGTAATGTTGGATGTGTTCCC
	GmUBI3-qR	ACACAATTGAGTTCAACACAAACCG
